# Supplementary material for: Predicting DNA structure using a deep learning method
Source: Nat Commun. 2024 Feb 9;15:1243. doi: 10.1038/s41467-024-45191-5 (PMC10858265; doi:10.1038/s41467-024-45191-5)
Supplement: Supplementary file 1 — Supplementary Information [file 41467_2024_45191_MOESM1_ESM.pdf]

SUPPLEMENTARY INFORMATION

Predicting DNA structure using a deep learning method

Jinsen Li[1], Tsu-Pei Chiu[1], and Remo Rohs[1,2,3,4,*]

[1]Department of Quantitative and Computational Biology, University of Southern California, Los Angeles, CA 90089, USA

[2]Department of Chemistry, University of Southern California, Los Angeles, CA 90089, USA

[3]Department of Physics and Astronomy, University of Southern California, Los Angeles, CA 90089, USA

[4]Thomas Lord Department of Computer Science, University of Southern California, Los Angeles, CA 90089, USA

*Correspondence: rohs@usc.edu

Supplementary Text

Training and evaluating Deep DNAshape on alternative datasets

In the manuscript, we discussed how to apply the Deep DNAshape model to DNA shape features derived from Monte-Carlo (MC) simulations. Based on our observation of Deep DNAshape performance on MC data, we concluded that the model architecture should extend to any data source. Therefore, we compiled an alternative dataset comprising DNA shape features obtained from experimentally solved structures, and a dataset with DNA shape features derived from a limited number of available MD simulations, to benchmark Deep DNAshape. We constructed the experimental dataset using a lightly curated list of PDB entries[1] that includes protein-bound DNA structures and irregular DNA structures, among others. We believe that our model architecture can efficiently address any existing biases and artifacts in this dataset. After processing (see Supplementary Methods), DNA shape features were normalized and used to train the Deep DNAshape model, resulting in the Deep DNAshape (Expt) variant. Concurrently, we used DNA shape data from the Parmbsc1 database[2], comprising only of B-DNA duplexes, to train another Deep DNAshape variant, Deep DNAshape (MD), despite the limited availability of MD data.

We compared DNA shape predicted by our MC-derived Deep DNAshape, Deep DNAshape (Expt), and Deep DNAshape (MD) models in several ways, assuming that the trained Deep DNAshape model can infer neighboring effects as accurately as possible. As the raw underlying data differ in sequence components, they could only be compared using constructed query tables, a method previously validated[3].

Initially, we directly compared the ability of the models to predict *k*-mers (minor groove width (MGW) and intra-base-pair (bp) feature predictions, on pentamers; inter-bp feature predictions on hexamers). We calculated correlations of the core DNA shape across all *k*-mers in the same order between models (Supplementary Table 3). We also compiled average inter-bp shape features across these models for the 10 unique dinucleotides (Supplementary Figure 4). The Deep DNAshape (Expt) variant performs almost identically compared to the underlying experimental PDB data. Deep DNAshape (MD) generally matches the experimental data, barring a slight overestimation of the feature Slide. Although Deep DNAshape underestimates Roll and Slide values compared to experimental data, the Roll values are better correlated with Deep DNAshape (Expt) compared to Deep DNAshape (MD) (Supplementary Table 3).

Next, we examined whether features predicted by the variants enhance the performance of the shape-augmented TF-DNA binding prediction models (multiple linear regression (MLR) models). Estimating longer-range neighboring effects is quite challenging with noisy or low-coverage data, even with Deep DNAshape's comprehensive control of overfitting. We compared performances of 1mer+4shape and

1mer+13shape MLR models (Supplementary Figure 22). Up to shape layer 4, the performances of Deep DNAshape (Expt), Deep DNAshape (MD), and Deep DNAshape (MC) are statistically indistinguishable. However, when the layer number further increases, the performance of Deep DNAshape remains constant, whereas the performances of the other two variants exhibit changes (MD in 1mer+4shape model and Expt in both models). As per the design of the Deep DNAshape architecture, early layers do not encompass all long-range neighboring effects, meaning we should focus on layers 3, 4, and 5. At layer 5, Deep DNAshape outperforms variants trained with other data sources. MD simulations also provide the capacity to learn DNA shape fluctuations. However, the performance of Deep DNAshape (MD) for the 1mer+4shape+FL model suffers dramatically in deeper layers (Supplementary Figure 22), likely due to the low coverage of the underlying MD data.

Limitations and Discussion

Successful training of Deep DNAshape on structural data acquired from experimentally solved structures and MD simulations confirms the generality and robustness of the Deep DNAshape model architecture. However, experimentally solved structures contain long DNA sequences bound by proteins only in certain circumstances, possibly deforming the DNA structures to a certain extent. An example is the nucleosome structure[4] of which many different copies exist in the PDB. This causes Deep DNAshape (Expt) models to learn incorrect neighboring effects, leading to worse performance in the deeper layers. Furthermore, available data from MD simulations is currently still limited with many missing values (Supplementary Figure 8), causing Deep DNAshape (MD) to learn incomplete neighboring effects from these missing values. Use of MC simulations as our underlying shape source represents at this point a favorable balance of data quality and quantity. Transitioning to MD simulations for training Deep DNAshape will likely become an alternative in the future, provided a sufficiently larger number of MD simulations could be procured.

Supplementary Methods

Acquisition, analysis of experimentally solved structures, and pre-processing for Deep DNAshape (Expt)

Biological assemblies of PDB IDs mentioned in reference[1] were downloaded from the PDB. Duplex DNA helical sections were extracted from all PDB files using X3DNA[5]. DNA shape features were calculated using Curves (version 5.3), where applicable, resulting in a total of 3,204 PDB IDs included (4,034 for biological assemblies). Some bases for which DNA shape could not be computed resulted in NaN values. Numerical shape features in regions containing the abnormal DNA helix were set to NaN. Criteria were based on the original DNAshape paper[6], in which regions were disregarded if one of the following conditions existed within 3 bp: (i) MGW > 8.5 Å or < 1.5 Å; (ii) HelT > 45°; and (iii) |Roll| > 20°. Chemically modified bases were converted to the original regular base in the final file. Any other irregular bases present in the dataset were set to unknown (N). Shape features of both strands were extracted. Calculated DNA shape features, including features of the reverse complements, were merged. If more than one copy of DNA exists, the values were averaged and NaN values were ignored. This protocol was adopted from[6].

Structures may have very short DNA sequences resulting in more incomputable groove or inter-bp features than intra-bp features. After preprocessing these structures and merging repeated sequences, we collected 2,129 sequences of available MGW values (about 15.47 bp per sequence), 2,269 sequences of available inter-bp shape feature values (about 15.02 bp per sequence) and 2,367 sequences of available intra-bp shape features (about 14.89 bp per sequence). For comparison purposes, average inter-bp shape features were calculated from this training data for each of the 10 unique dinucleotides.

Compiled shape training data were then pre-processed using normalization (Methods). However, due to volatility of the dataset, we adjusted the percentile from 1 to 5:

$$\hat{S} = (S - \tilde{S})/(S_{5th} - S_{95th}) \quad (1)$$

Here, S represents the DNA shape feature analyzed from the experimentally solved structural dataset. $\tilde{S}$ denotes the median of the DNA shape feature values within the dataset, while S_{5th} and S_{95th} mark the 5th percentile and 95th percentile of the sorted DNA shape feature values, respectively.

Acquisition of MD simulation DNA shape data

DNA shape data files, labeled with tags of "DNA" (DNA only) and "duplex", were downloaded from <https://mmb.irbbarcelona.org/ParmBSC1/>. Files were merged and aggregated with the addition of reverse complements. We collected 137 sequences for MGW (about 15.89 bp per sequence), 139 sequences containing inter-bp features (about 15.92 bp per sequence) and 138 sequences containing intra-bp features (about 15.92 bp per sequence).

Supplementary Tables and Figures

Supplementary Table 1. Correlation of inter-bp features predicted by Deep DNAshape and acquired from MD simulations, for all 136 unique tetramers[7]. Tetramer features from Deep DNAshape are calculated by predicting and averaging from all 8-mers.

Inter-bp features	Spearman's rank correlation of static shape	Spearman's rank correlation of shape fluctuation
Shift	0.23	0.22
Slide	0.51	0.53
Rise	0.41	0.78
Tilt	0.59	0.85
Roll	0.79	0.32
HelT	0.44	0.41

Supplementary Table 2. Pearson's correlations of DNA shape features predicted by Deep DNAshape and reconstructed query table from MC simulations. Reconstructed hexamer and 7-mer query tables contain extensive missing values. There are 10 possible dinucleotides. We show NpN in this table for easier representation. However, the generated query table for hexamers does not account for all dinucleotides; hence, the lower correlation. The pentamer query table came with the DNAshape method. "Core" means the central bp or bp step from the *k*-mer query table.

<table>
<tr><th rowspan="2">Shape feature</th><th>Against tetramer</th><th colspan="2">Against pentamer</th><th>Against hexamer</th><th colspan="2">Against heptamer</th></tr>
<tr><th>Core: NpN</th><th>Core: A/T</th><th>Core: C/G</th><th>Core: NpN</th><th>Core: A/T</th><th>Core: C/G</th></tr>
<tr><td>MGW</td><td>N/A</td><td>0.98</td><td>0.98</td><td>N/A</td><td>0.96</td><td>0.96</td></tr>
<tr><td>Shift</td><td>0.99</td><td colspan="2" rowspan="6">N/A</td><td>0.82</td><td colspan="2" rowspan="6">N/A</td></tr>
<tr><td>Slide</td><td>0.98</td><td>0.87</td></tr>
<tr><td>Rise</td><td>0.99</td><td>0.97</td></tr>
<tr><td>Tilt</td><td>0.99</td><td>0.78</td></tr>
<tr><td>Roll</td><td>0.98</td><td>0.68</td></tr>
<tr><td>HelT</td><td>1.00</td><td>0.91</td></tr>
<tr><td>Shear</td><td rowspan="6">N/A</td><td>0.97</td><td>0.96</td><td rowspan="6">N/A</td><td>0.94</td><td>0.92</td></tr>
<tr><td>Stretch</td><td>0.78</td><td>0.94</td><td>0.77</td><td>0.87</td></tr>
<tr><td>Stagger</td><td>0.98</td><td>0.99</td><td>0.97</td><td>0.98</td></tr>
<tr><td>Buckle</td><td>0.99</td><td>0.99</td><td>0.98</td><td>0.99</td></tr>
<tr><td>ProT</td><td>0.99</td><td>1.00</td><td>0.97</td><td>0.98</td></tr>
<tr><td>Opening</td><td>0.93</td><td>0.97</td><td>0.87</td><td>0.92</td></tr>
</table>

Supplementary Table 3. Pearson's correlations of DNA shape features predicted by Deep DNAshape and its variants. All unique pentamers (512 sequences) and hexamers (2,080 sequences) are used to predict DNA shape. Correlations are calculated by the concatenated predictions as a vector given the same order of sequences.

[See Supplementary_Table3.xlsx]

Supplementary Table 4. Pearson's correlations of average DNA shape predicted by Deep DNAshape or pentamer-based DNAshape for four different *Drosophila* species.

MGW derived from Deep DNAshape

Species	*D. melanogaster*	*D. simulans*	*D. sechellia*	*D. pseudoobscura*
D. melanogaster	1.00	0.95	0.96	0.93
D. simulans		1.00	0.99	0.97
D. sechellia			1.00	0.97
D. pseudoobscura				1.00

MGW derived from pentamer-based DNAshape

Species	*D. melanogaster*	*D. simulans*	*D. sechellia*	*D. pseudoobscura*
D. melanogaster	1.00	0.93	0.91	0.91
D. simulans		1.00	0.97	0.95
D. sechellia			1.00	0.95
D. pseudoobscura				1.00

ProT derived from Deep DNAshape

Species	*D. melanogaster*	*D. simulans*	*D. sechellia*	*D. pseudoobscura*
D. melanogaster	1.00	0.94	0.95	0.91
D. simulans		1.00	0.98	0.95
D. sechellia			1.00	0.95
D. pseudoobscura				1.00

ProT derived from pentamer-based DNAshape

Species	*D. melanogaster*	*D. simulans*	*D. sechellia*	*D. pseudoobscura*
D. melanogaster	1.00	0.93	0.90	0.89
D. simulans		1.00	0.96	0.94
D. sechellia			1.00	0.93
D. pseudoobscura				1.00

Roll derived from Deep DNAshape

Species	*D. melanogaster*	*D. simulans*	*D. sechellia*	*D. pseudoobscura*
D. melanogaster	1.00	0.97	0.98	0.97
D. simulans		1.00	0.99	0.99
D. sechellia			1.00	0.98
D. pseudoobscura				1.00

Roll derived from pentamer-based DNAshape

Species	*D. melanogaster*	*D. simulans*	*D. sechellia*	*D. pseudoobscura*
D. melanogaster	1.00	0.94	0.95	0.94
D. simulans		1.00	0.97	0.97
D. sechellia			1.00	0.96
D. pseudoobscura				1.00

HelT derived from Deep DNAshape

Species	*D. melanogaster*	*D. simulans*	*D. sechellia*	*D. pseudoobscura*
D. melanogaster	1.00	0.94	0.96	0.95

D. simulans		1.00	0.98	0.96
D. sechellia			1.00	0.96
D. pseudoobscura				1.00

HelT derived from pentamer-based DNAshape

Species	*D. melanogaster*	*D. simulans*	*D. sechellia*	*D. pseudoobscura*
D. melanogaster	1.00	0.93	0.91	0.93
D. simulans		1.00	0.95	0.94
D. sechellia			1.00	0.94
D. pseudoobscura				1.00

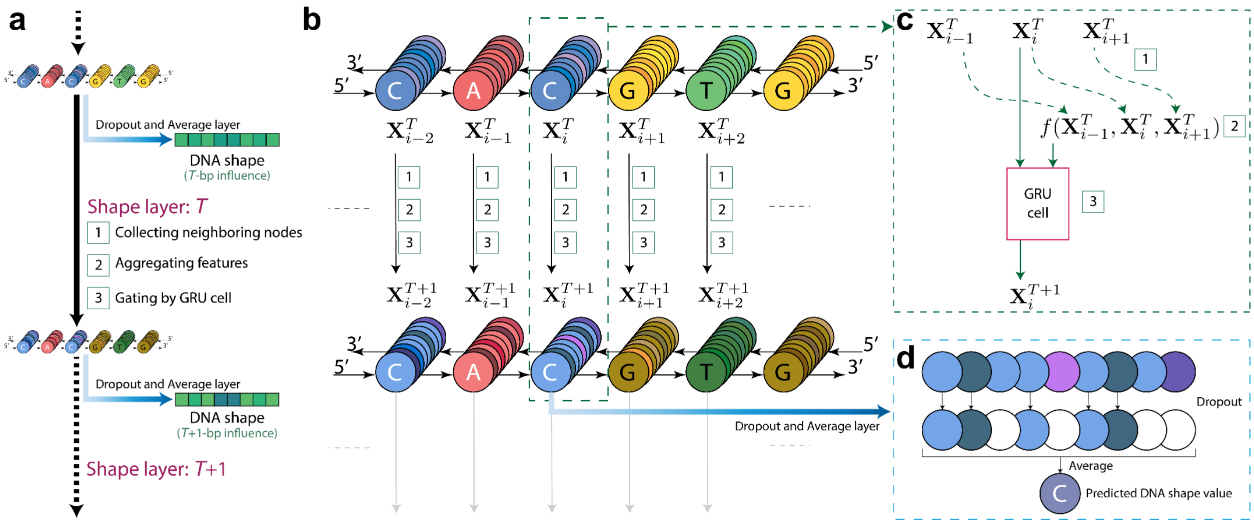

Supplementary Figure 1. Deep DNAshape architecture.

a) Overview of shape layer. Features (stored in nodes) are used as input to the shape layer. Before the shape layer, nodes are first transformed from one-hot encoding of mono- or dinucleotides through one self-convolution layer into features. Nodes are interconnected through edges in the data structure. New features (stored in nodes, considering the two neighboring nodes) are the output of the shape layer.

b) Detailed parallel computation schema for shape layer.

c) Calculation in shape layer. Features from neighboring nodes are collected, aggregated by trainable equation (see Methods), and gated by a trainable GRU cell to generate the new feature.

d) Dropout and average layer for DNA shape output.

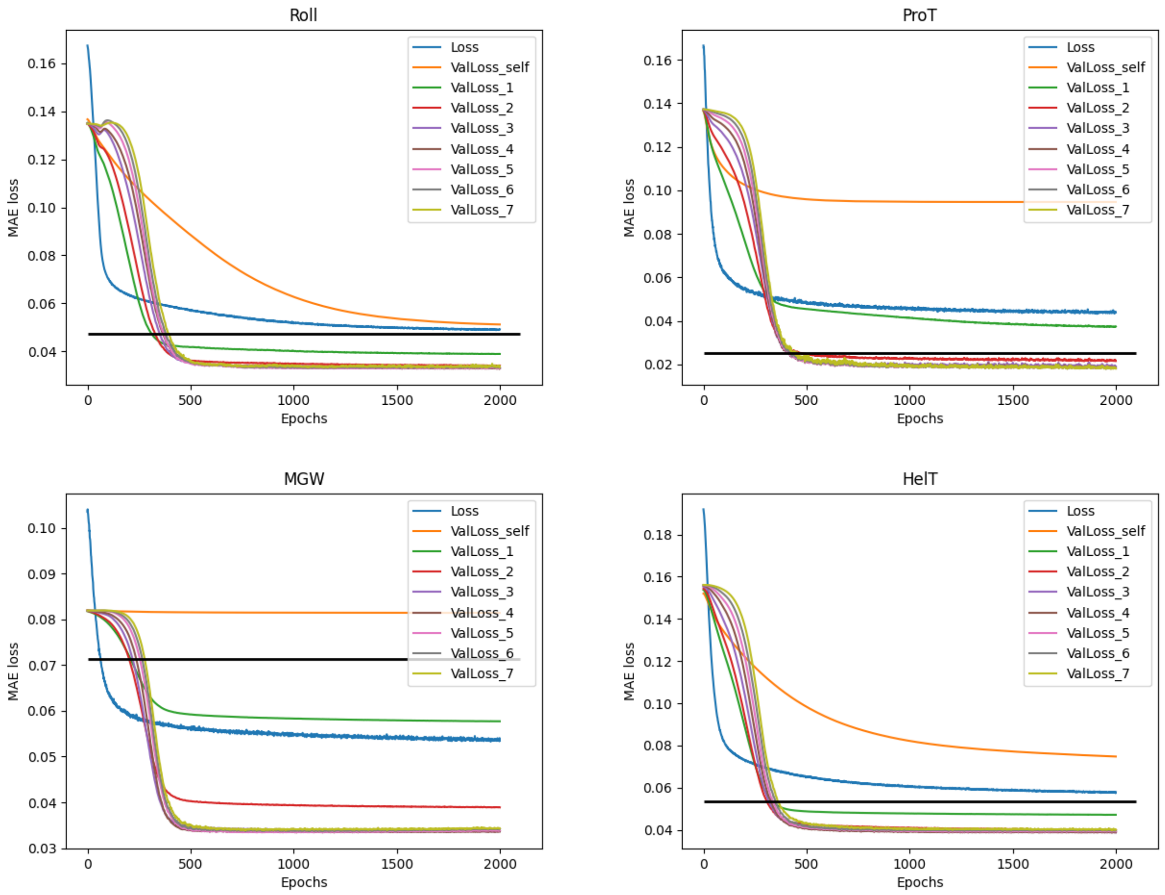

Supplementary Figure 2. Training curves for four DNA shape features (Roll, ProT, MGW, and HelT), for a training and validation split (80/20 split). "Loss" represents training loss of mean absolute error (MAE). "ValLoss" represents the MAE loss on validation set. "self" and "1" to "7" mean different layers outputted from the model. Black horizontal line is a reference loss, as if the same validation data were to be predicted by the pentamer-based DNAshape method[3].

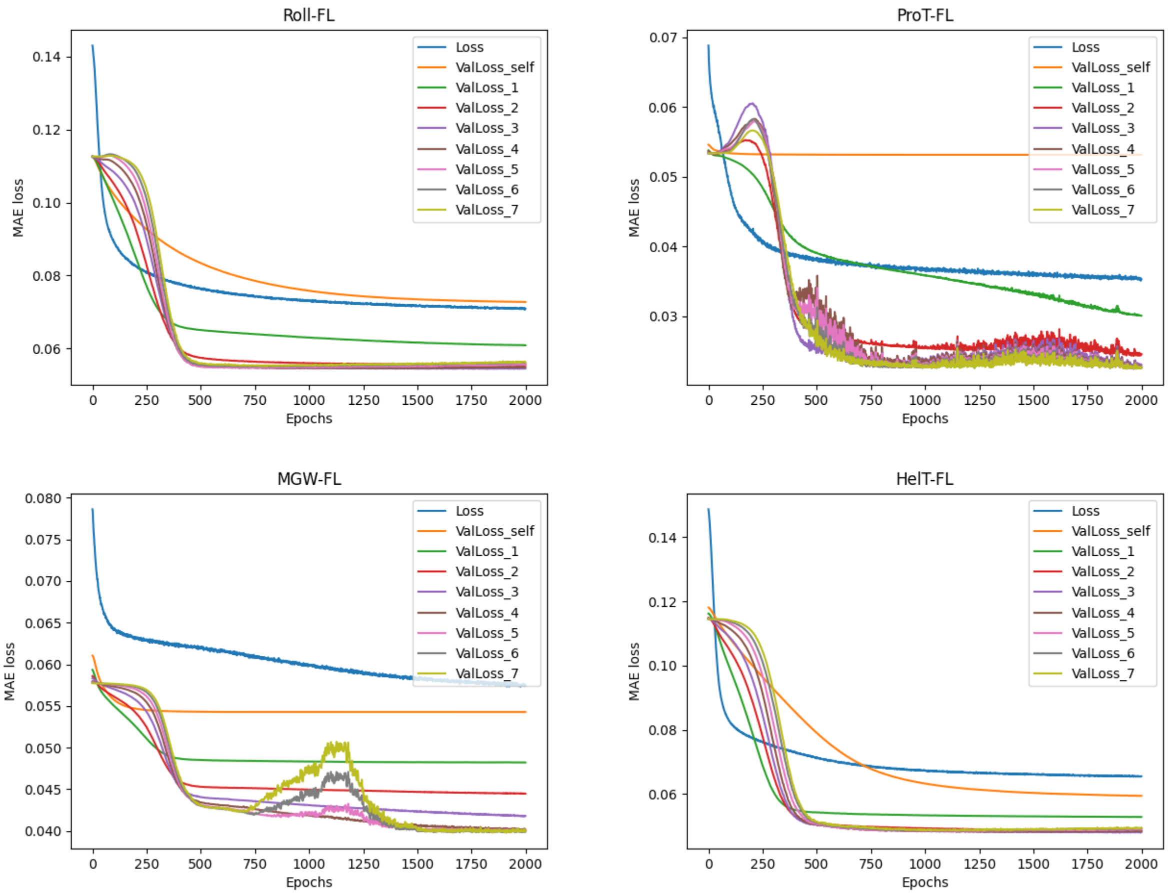

Supplementary Figure 3. Training curves for four DNA shape (Roll, ProT, MGW, and HelT) fluctuation values, for a training and validation split (80/20 split). "Loss" represents the training loss of MAE. "ValLoss" represents the MAE loss on validation set. "self" and "1" to "7" mean different layers outputted from the model. Reference from the pentamer-based DNAshape method does not exist.

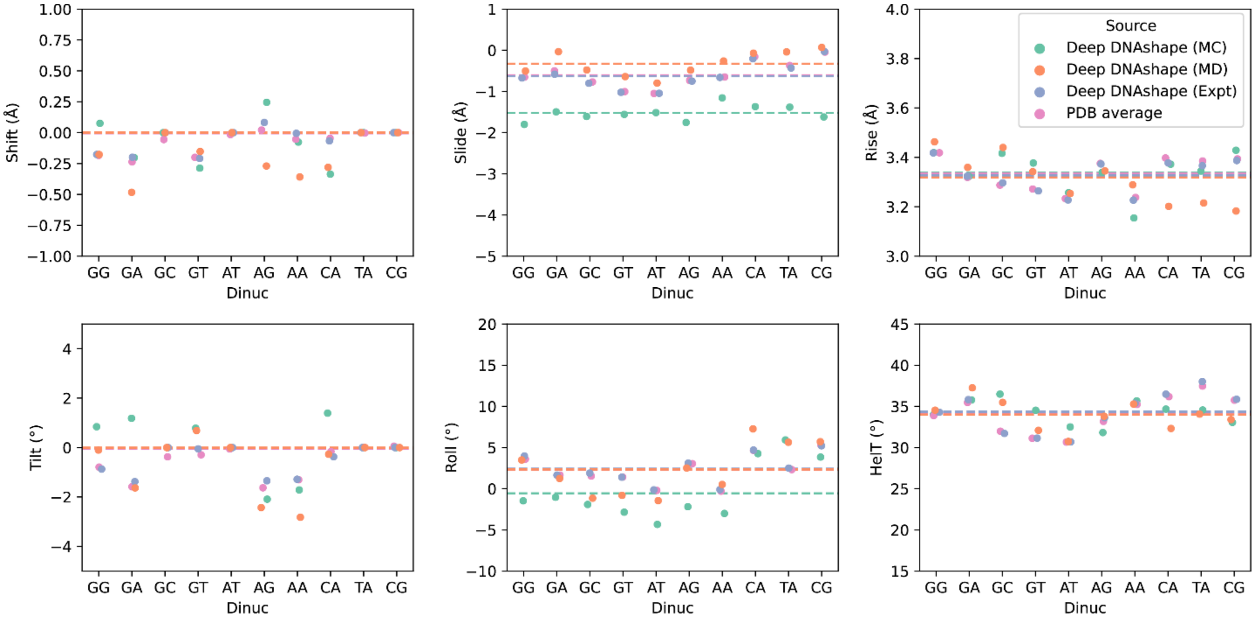

Supplementary Figure 4. Averaged inter-base-pair shape features for the 10 dinucleotides.

Shown are DNA shape features of all hexamers (2,080 sequences) predicted by the Deep DNAshape model and its variants, layer 2. PDB averages are calculated from filtered PDB entries (see Supplementary Methods). Horizontal lines are averaged values among the 10 dinucleotides for each model. Slide and Roll values are underestimated by Deep DNAshape (MC) compared to PDB average. Slide values are overestimated by Deep DNAshape (MD). Values are jittered horizontally to reveal minor difference. Correlations of the 2,080 hexamers can be found in Table S3.

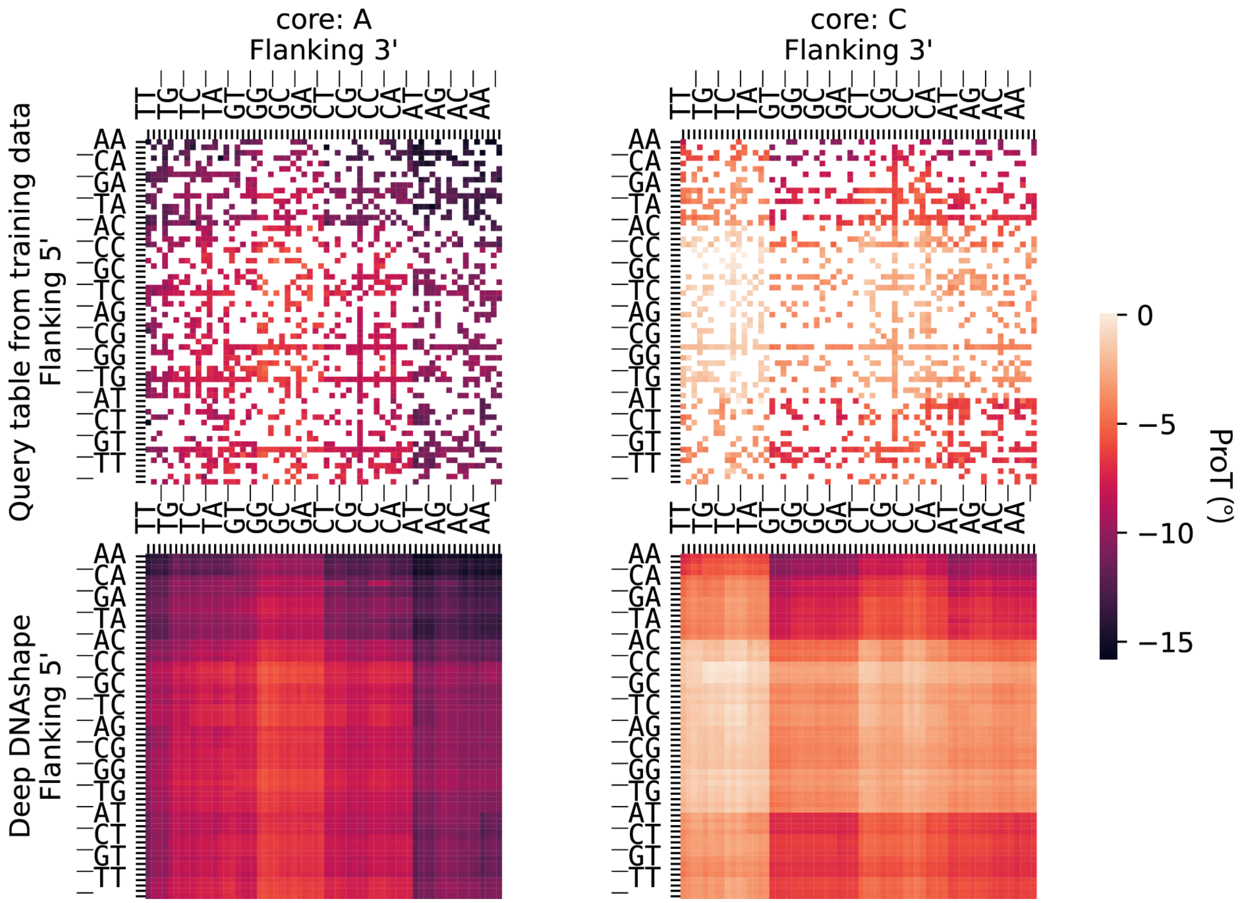

Supplementary Figure 5. Heatmaps showing ProT values at central position for all possible 7-mers. Upper two subpanels show ProT values as if we had constructed a 7-mer query table from available MC simulations directly. Lower two subpanels show ProT values predicted by Deep DNAshape method (layer 3) for all possible 7-mers. Left two subpanels show all sequence with 'A' base in the center. Right two subpanels show all sequences with 'C' base in the center. '_' represents A, C, G, and T in sequential order. For example, top left grid represents ProT value for AAAATTT in the central position.

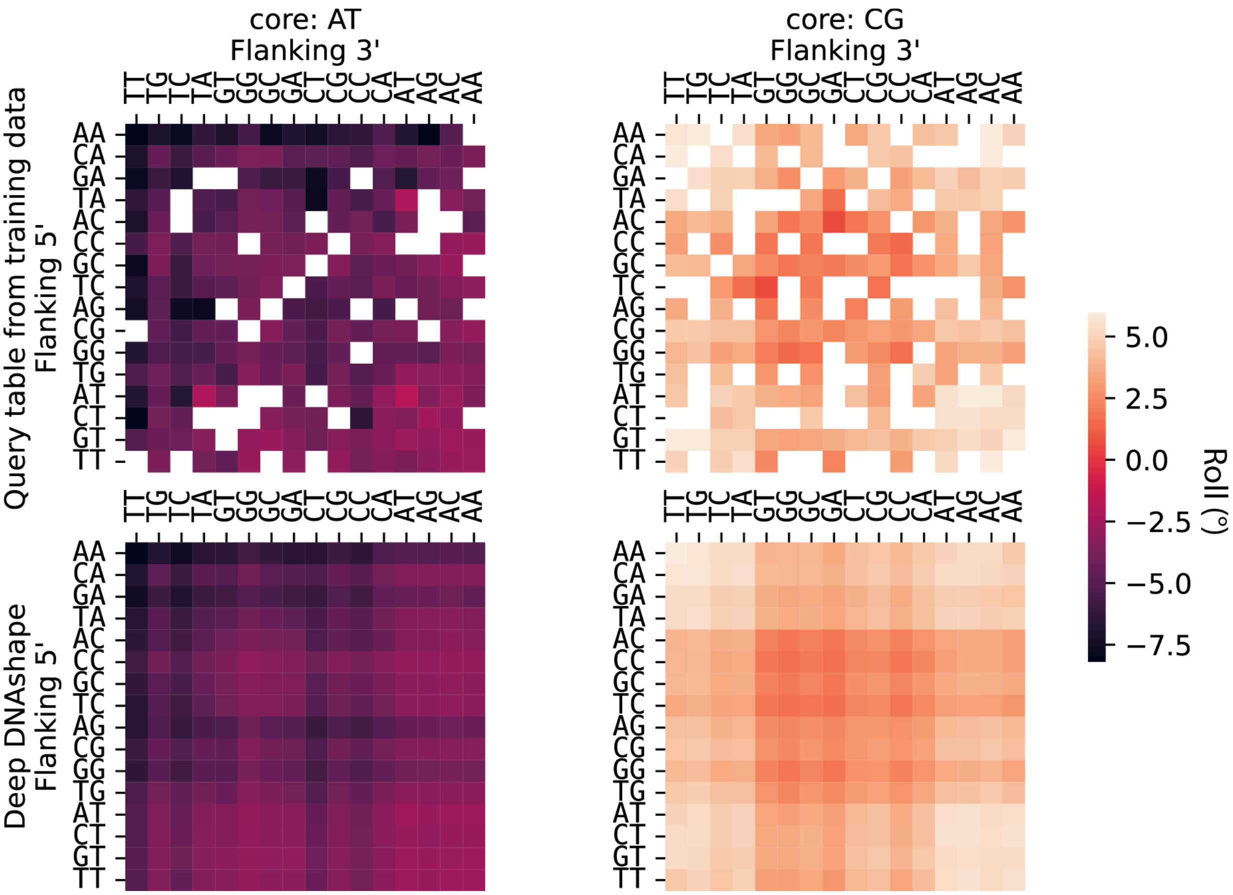

Supplementary Figure 6. Heatmaps showing Roll values at central position for all possible 6-mers. Upper two subpanels show Roll values as if we had constructed a 6-mer query table from available MC simulations directly. Lower two subpanels show Roll values predicted by the Deep DNAshape method (layer 3) for all possible 6-mers. Left two subpanels show all sequences with 'AT' dinucleotides in the center. Right two subpanels show all sequences with 'CG' dinucleotides in the center. For example, top left grid represents Roll value for AAATTT in the central position.

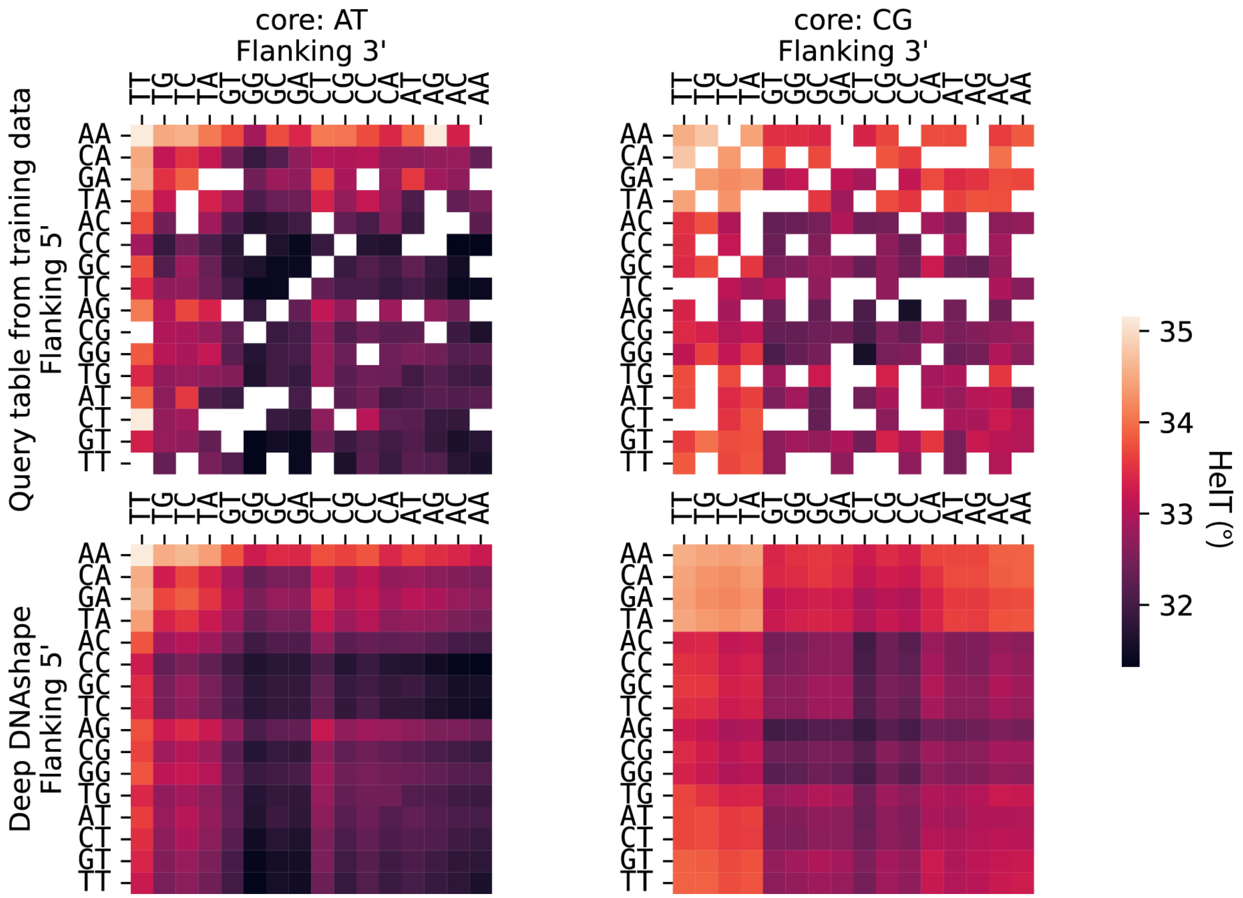

Supplementary Figure 7. Heatmaps showing HelT values at central position for all possible 6-mers. Upper two subpanels show HelT values as if we had constructed a 6-mer query table from available MC simulations directly. Lower two subpanels show HelT values predicted by Deep DNAshape method (layer 2) for all possible 6-mers. Left two subpanels show all sequences with 'AT' dinucleotides in middle. Right two subpanels show all sequences with 'CG' dinucleotides in middle. For example, top left grid represents HelT value for AAATTT in the central position.

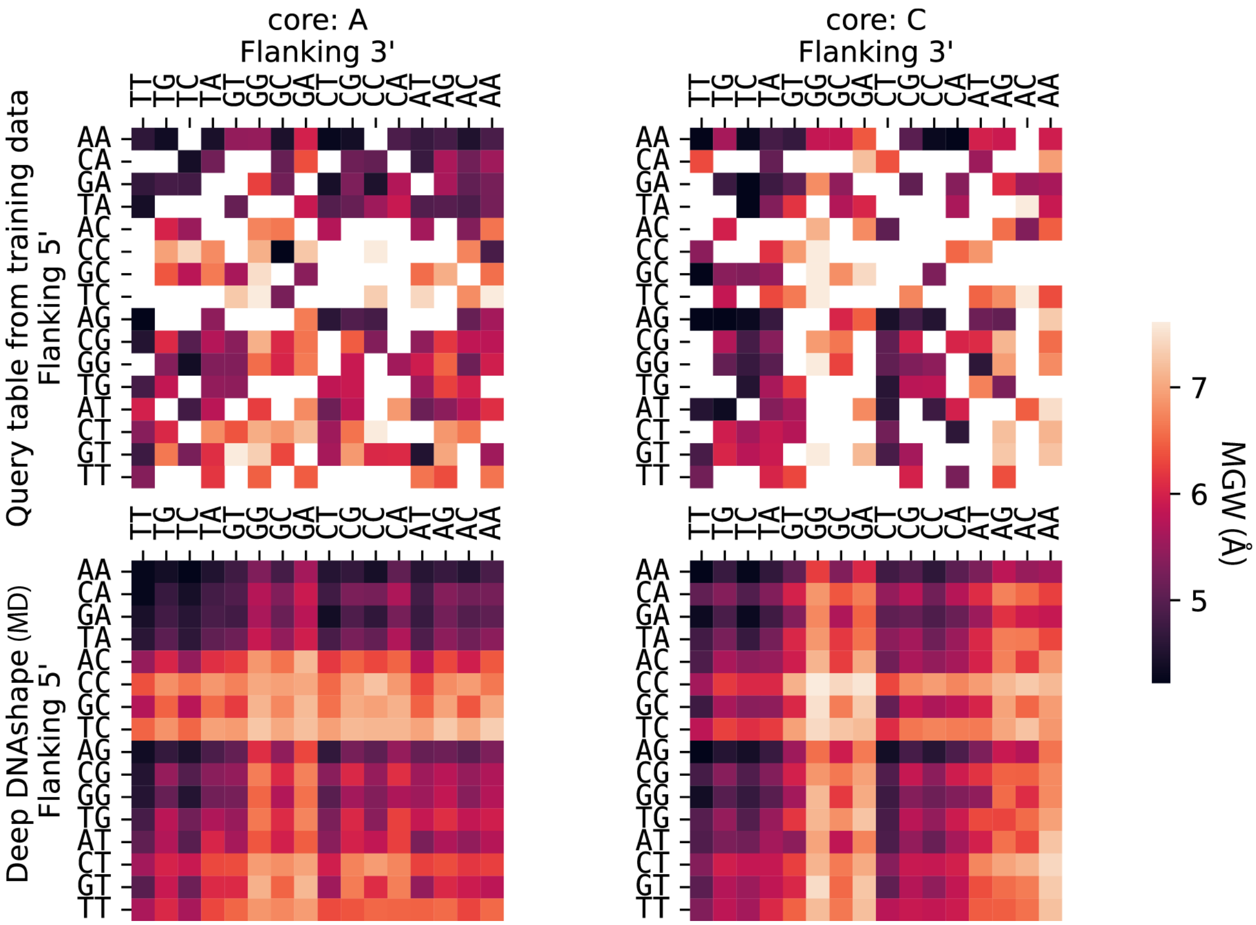

Supplementary Figure 8. MD version of heatmaps showing MGW values at central position for all possible 5-mers. Upper two subpanels show MGW values as if we had constructed a 5-mer query table from available MD simulations directly. Lower two subpanels show MGW values predicted by Deep DNAshape (MD) method (layer 2) for all possible 5-mers. Left two subpanels show all sequences with ‘A’ base in the center. Right two subpanels show all sequences with ‘C’ base in the center.

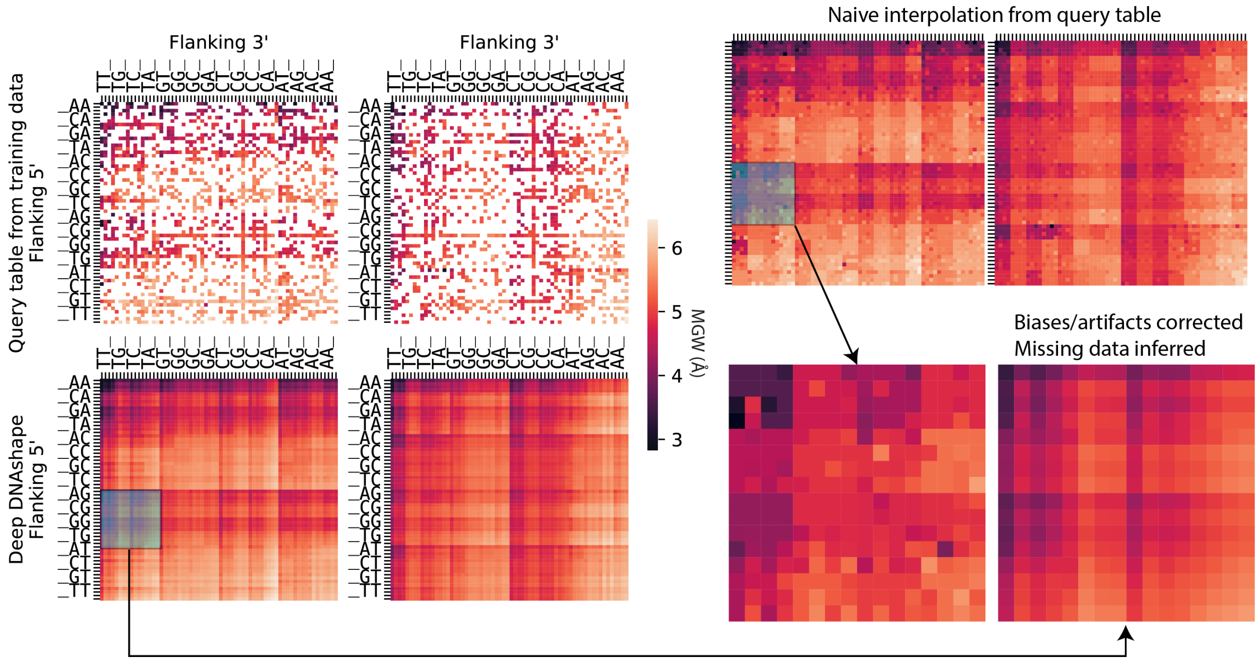

Supplementary Figure 9. Comparison between naïve interpolation and Deep DNAshape for generating heptamer query table from training data. Patch showing MGW for all possible NNGATNN (N can be any of A, C, G, or T) sequences is selected for comparison in right bottom panels. Deep DNAshape provides detailed, unbiased information on effects of flanking regions compared to interpolated version.

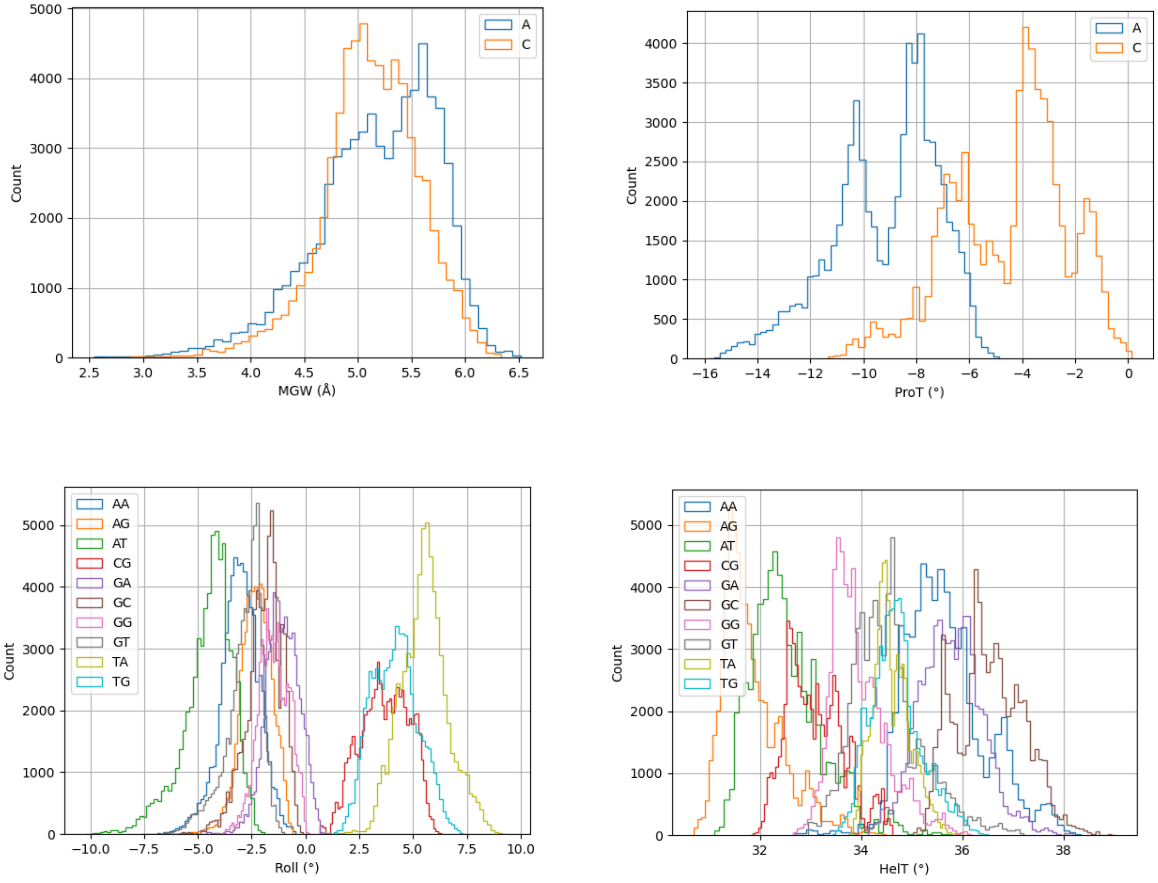

Supplementary Figure 10. Predicted DNA shape features for cores (shown in legend), affected by all possible 4-bp of flanking regions. Features are predicted by Deep DNAshape method (layer 4). MGW is a groove feature assigned to mononucleotides, and ProT is an intra-bp feature. Roll and HelT are inter-bp features assigned to dinucleotides. Histograms are generated from all possible 9-mers for MGW and ProT, 10-mers for Roll and HelT.

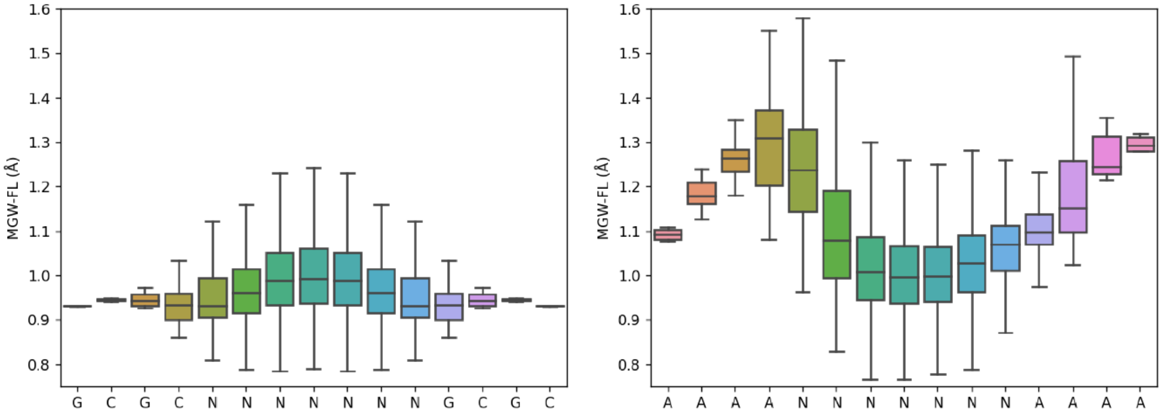

Supplementary Figure 11. MGW-FL (minor groove width fluctuations) predicted by Deep DNAshape (layer 4) for all possible random sequences with fixed 5' and 3' caps. Left panel is capped by 'GCGC'. Right panel is capped by 'AAAA'. Center line indicates the median. Box limits are 75th and 25th percentiles. The whiskers extend 1.5 times the IQR from the top and bottom of the box. Outliers are removed in boxplots. Number of samples is 16,384 for all combinations of 7-mers.

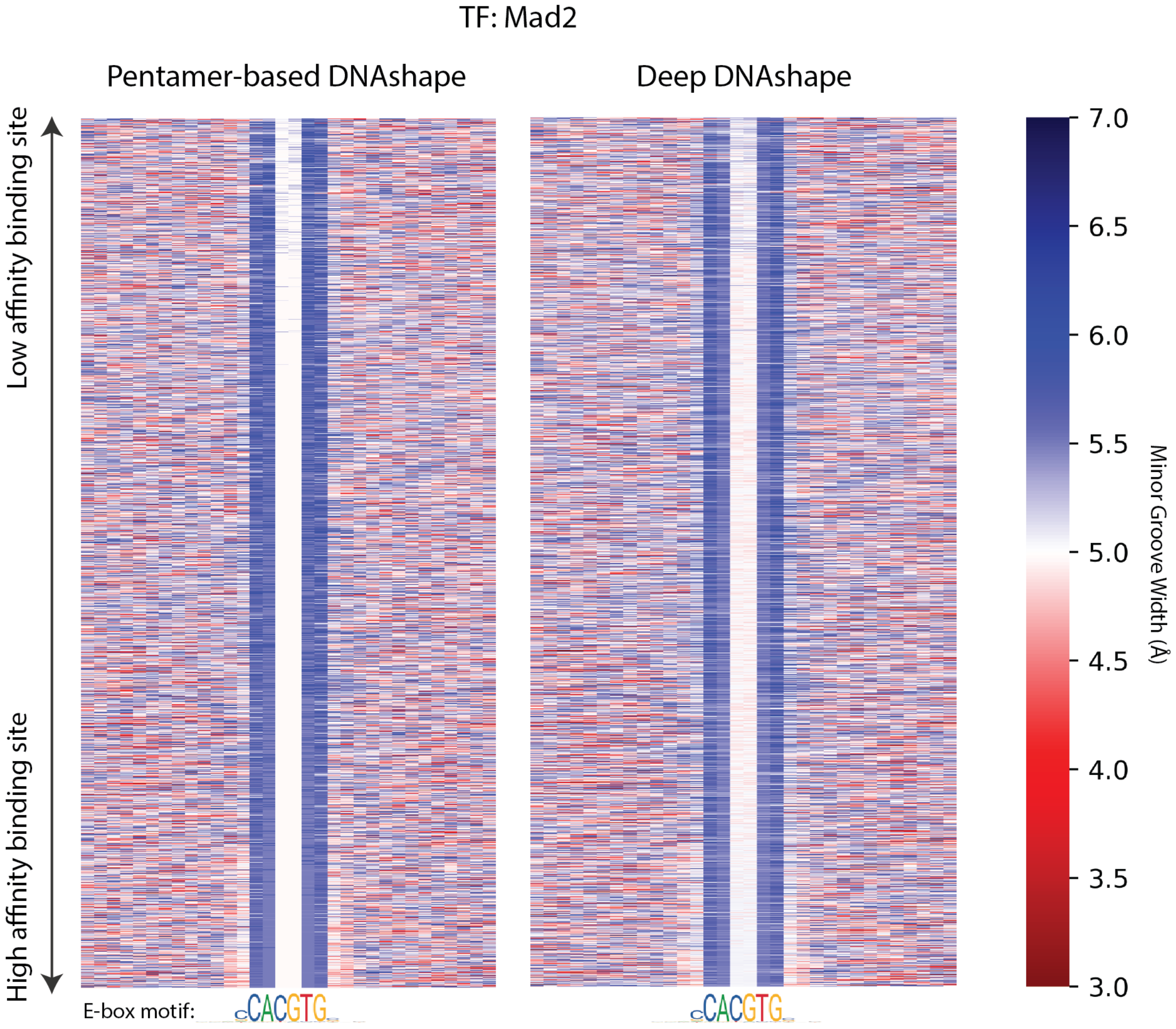

Supplementary Figure 12. MGW predicted by pentamer-based DNAshape or Deep DNAshape (layer 4), for protein Mad2 (Mad2-Max heterodimer) and DNA binding data, in order of relative binding affinity. Color represents DNA shape values. Data are aligned with core binding site. Only top 25% of binding data are used.

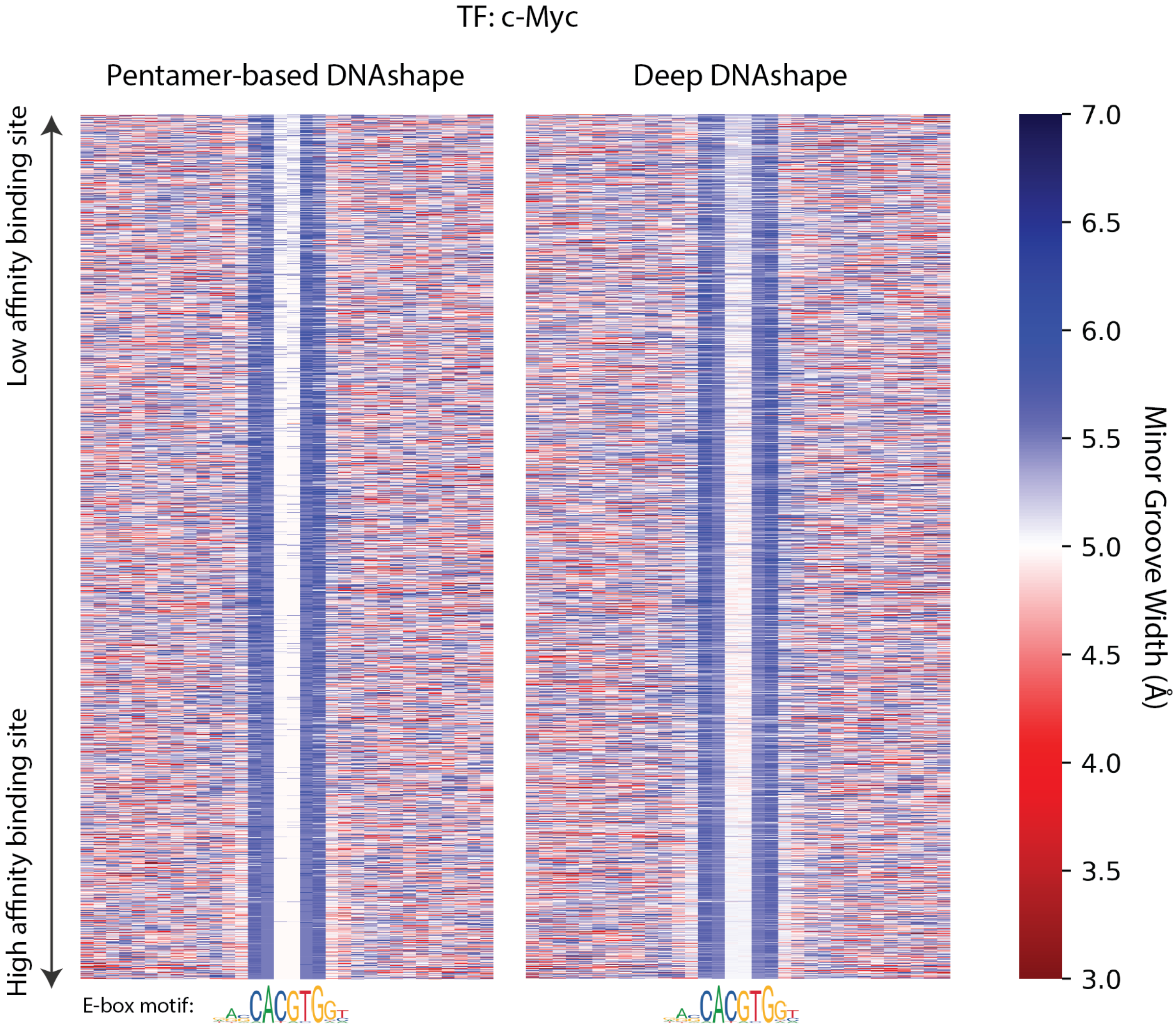

Supplementary Figure 13. MGW values predicted by pentamer-based DNAshape or Deep DNAshape (layer 4), for protein c-Myc (c-Myc-Max heterodimer) and DNA binding data, in order of relative binding affinity. Color represents DNA shape values. Data are aligned with core binding site. Only top 25% of binding data are used.

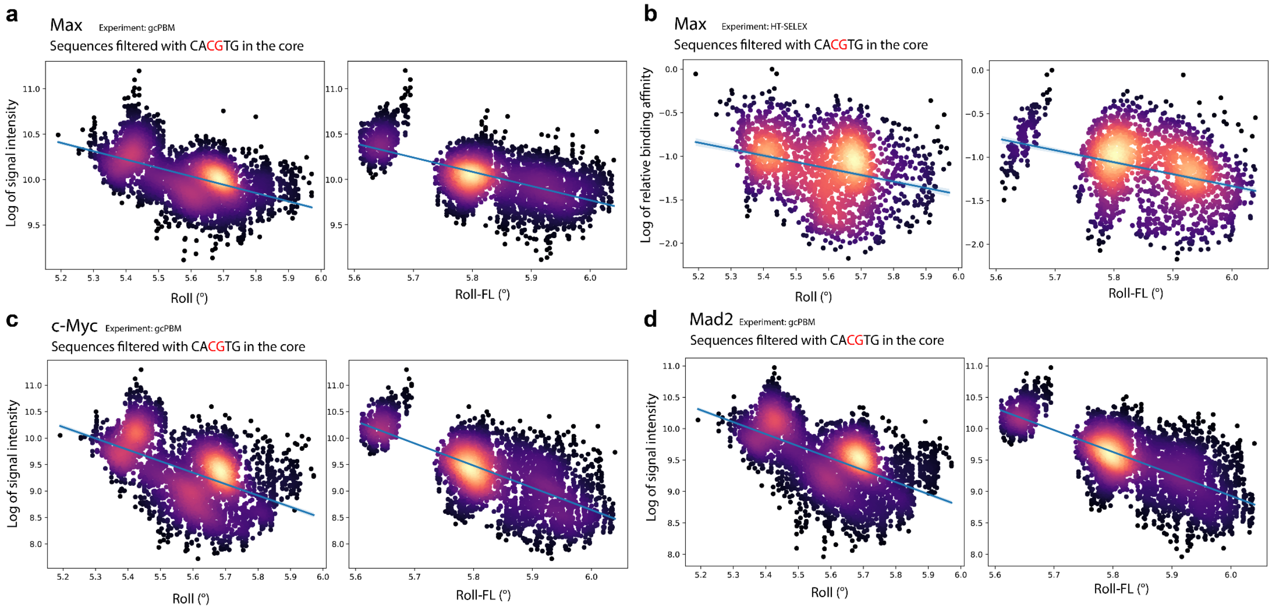

Supplementary Figure 14. Predicted Roll and Roll-FL values for Max, c-Myc and Mad2 binding data, compared to relative binding affinities from gcPBM and HT-SELEX experiment. Data is filtered with 'CACGTG' as the core region. Roll and Roll-FL features are predicted by Deep DNAshape, layer 5, for the central 'CG' bp step. Negative correlation can be found in these features.

a) Max homodimer data from gcPBM experiment.
b) Max homodimer data from HT-SELEX experiment.
c) c-Myc-Max heterodimer data from gcPBM experiment.
d) Mad2-Max heterodimer data from gcPBM experiment.

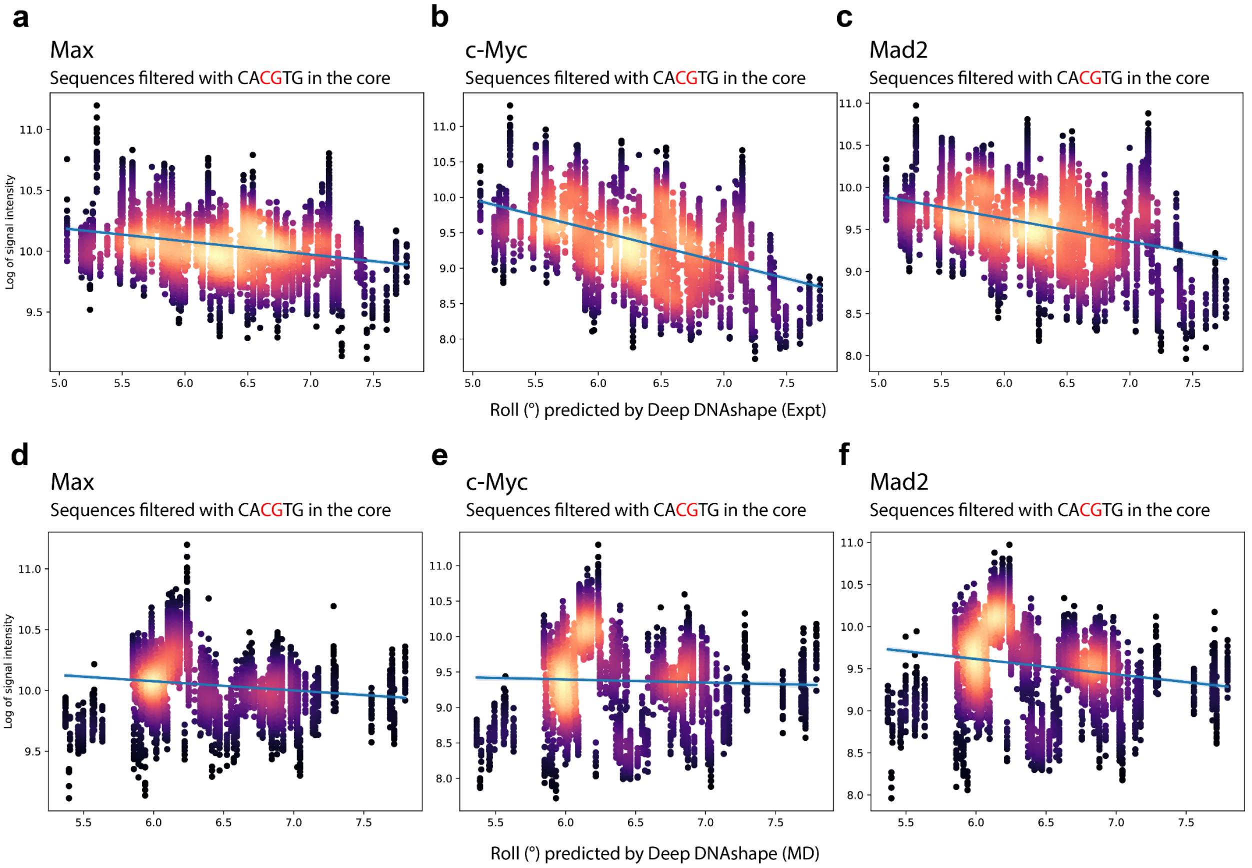

Supplementary Figure 15. Predicted Roll values for Max, c-Myc, and Mad2 binding data from gcPBM experiments, compared to the signal intensity (relative binding affinity in gcPBM).

a-c) Roll shape features are predicted by Deep DNAshape (Expt), layer 4. Negative correlation can be found in these features.

b-f) Roll shape features are predicted by Deep DNAshape (MD), layer 4.

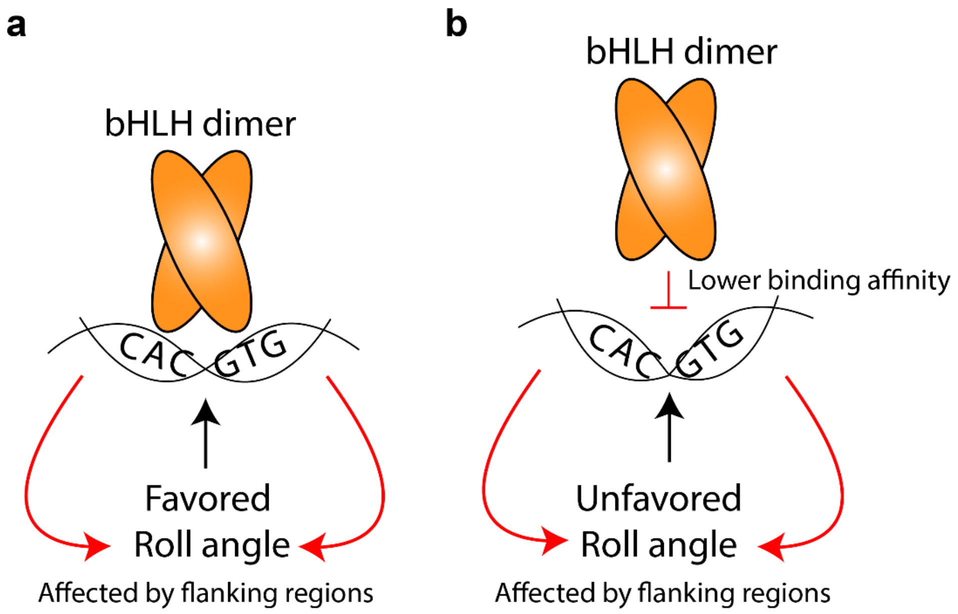

Supplementary Figure 16. Proposed binding mode of bHLH dimer using Roll readout to distinguish the same high-affinity binding sites.

a) bHLH dimer binds to high-affinity E-box binding sites ‘CACGTG’, where Roll values in the core (affected by the flanking regions) are favored.

b) bHLH dimer binds to the same high-affinity E-box binding sites ‘CACGTG’ with lowered binding affinity, where Roll values in the core (affected by the flanking regions) are less favored.

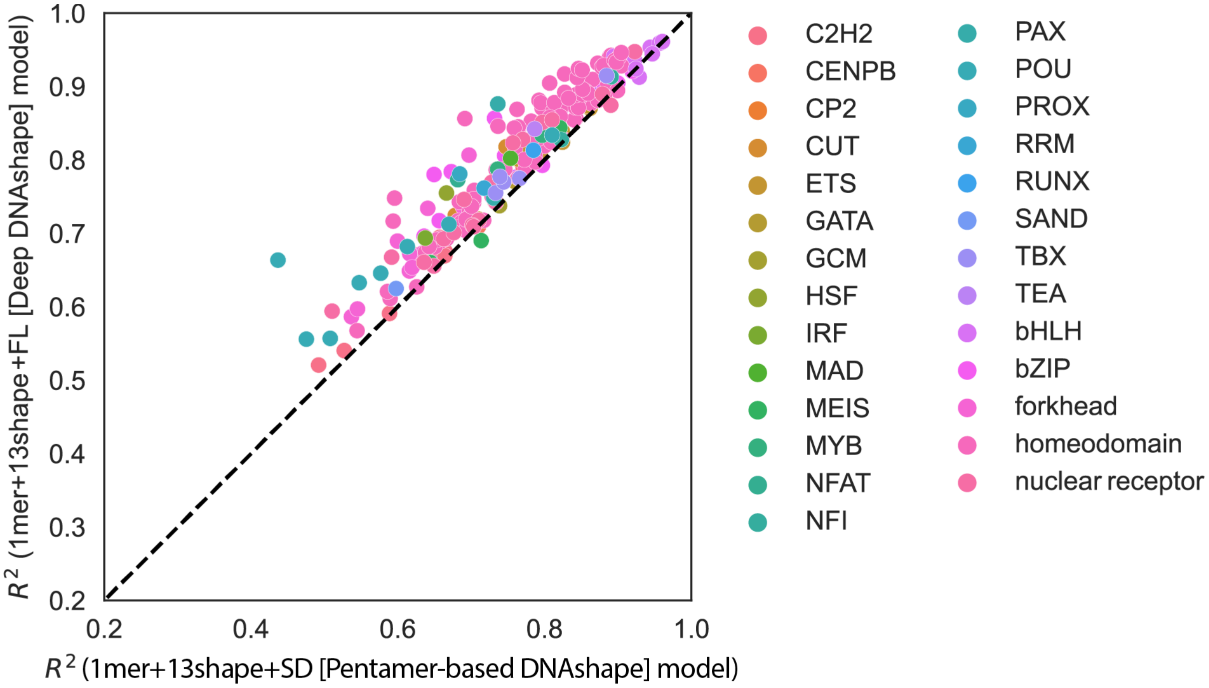

Supplementary Figure 17. R^2 comparison between 1mer+13shape+SD (pentamer-based DNAshape) and 1mer+13shape+FL (Deep DNAshape). SD values from pentamer-based DNAshape are calculated based on statistics when compiling the raw values in the simulation data. FL values predicted by Deep DNAshape are the real shape fluctuation values encountered during simulation.

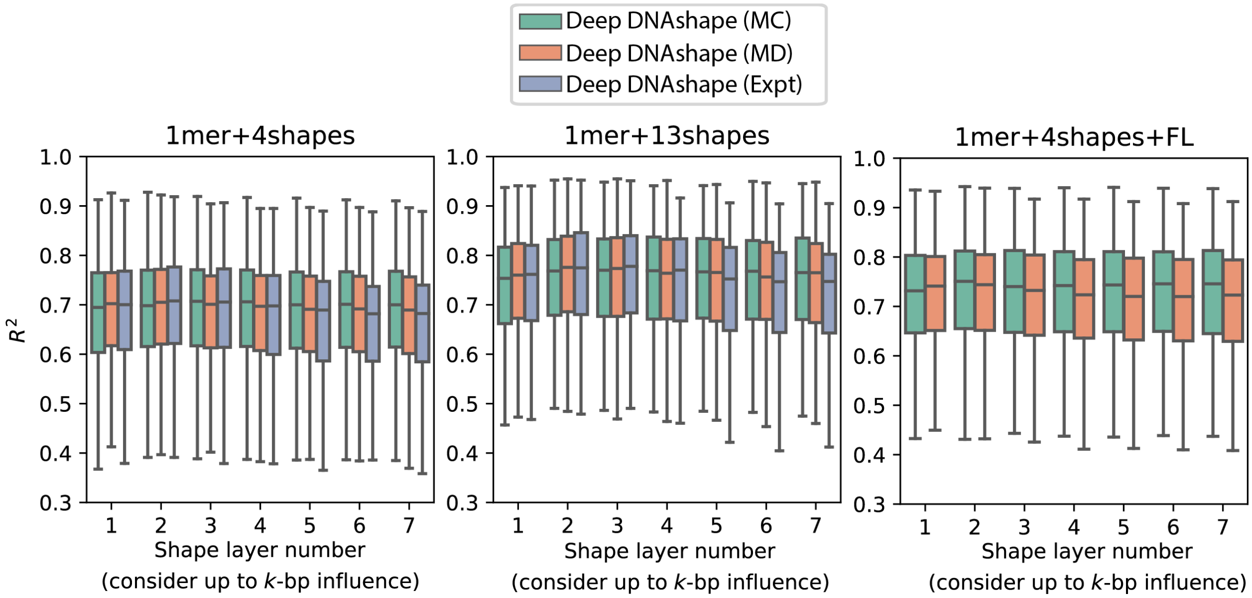

Supplementary Figure 18. R^2 performance of various MLR models on predicting collections of TF-DNA binding specificities from multiple experimental data.

Shape layer number indicates which depth of shape layer was used in the Deep DNAshape models to predict DNA shape features. Significant performance differences were only found in deeper layers, where Deep DNAshape on MC data nearly always showed the best performance. Center line indicates median. Box limits are 75th and 25th percentiles. Whiskers extend 1.5 times the inter-quartile range from the top and bottom of the box. Outliers are removed in boxplots. Number of TF-DNA data is 240 from gcPBM, SELEX-seq, and HT-SELEX datasets.

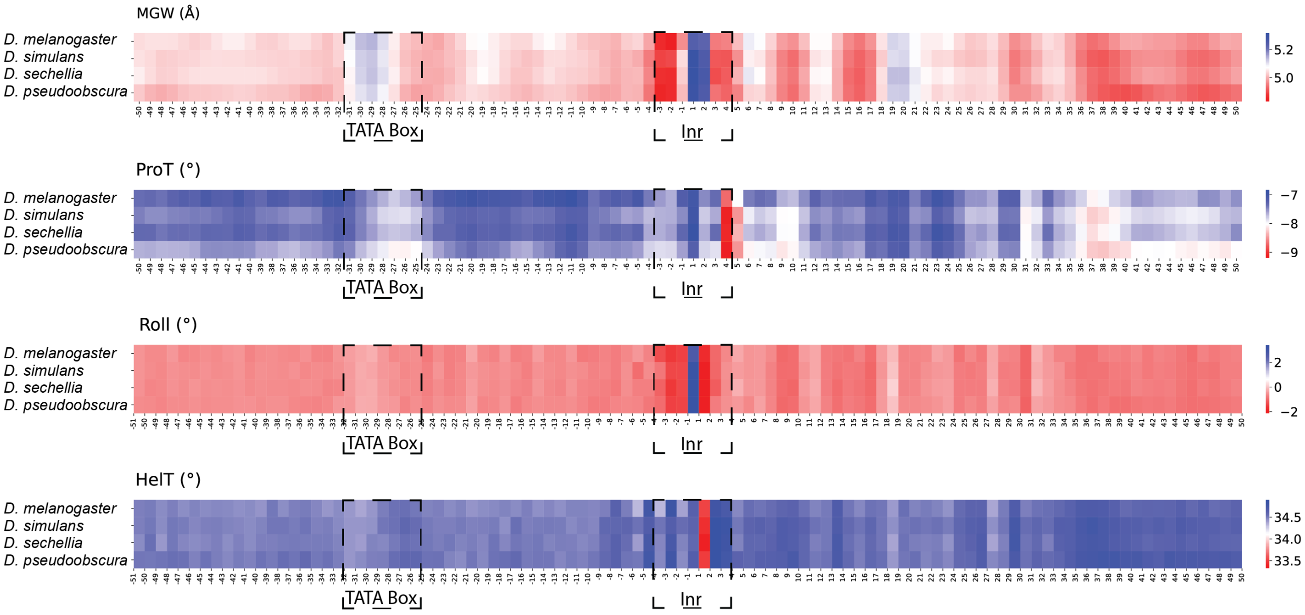

Supplementary Figure 19. Averaged DNA shape features predicted for transcription start sites (TSSs) from four different *Drosophila* species[8].

Supplementary References

1. Young, R. T., Czapla, L., Wefers, Z. O., Cohen, B. M. & Olson, W. K. Revisiting DNA sequence-dependent deformability in high-resolution structures: Effects of flanking base pairs on dinucleotide morphology and global chain configuration. *Life* **12**, 759 (2022).

2. Ivani, I. *et al.* Parmbsc1: A refined force field for DNA simulations. *Nat. Methods* **13**, 55–58 (2016).

3. Li, J. *et al.* Expanding the repertoire of DNA shape features for genome-scale studies of transcription factor binding. *Nucleic Acids Res.* **45**, 12877–12887 (2017).

4. Davey, C. A., Sargent, D. F., Luger, K., Maeder, A. W. & Richmond, T. J. Solvent mediated interactions in the structure of the nucleosome core particle at 1.9 Å resolution. *J. Mol. Biol.* **319**, 1097–1113 (2002).

5. Lu, X. J. & Olson, W. K. 3DNA: A software package for the analysis, rebuilding and visualization of three-dimensional nucleic acid structures. *Nucleic Acids Res.* **31**, 5108–5121 (2003).

6. Zhou, T. *et al.* DNAshape: A method for the high-throughput prediction of DNA structural features on a genomic scale. *Nucleic Acids Res.* **41**, W56-W62 (2013).

7. Pasi, M. *et al.* μABC: A systematic microsecond molecular dynamics study of tetranucleotide sequence effects in B-DNA. *Nucleic Acids Res.* **42**, 12272–12283 (2014).

8. Chiu, T. P. *et al.* GBshape: A genome browser database for DNA shape annotations. *Nucleic Acids Res.* **43**, D103-D109 (2015).
