# Supplementary material for: Predicting DNA structure using a deep learning method
Source: Nat Commun. 2024 Feb 9;15:1243. doi: 10.1038/s41467-024-45191-5 (PMC10858265; doi:10.1038/s41467-024-45191-5)
Supplement: Supplementary file 3 — Reporting Summary [file 41467_2024_45191_MOESM3_ESM.pdf]

nature portfolio

Corresponding author(s): Remo Rohs

Last updated by author(s): Jan 5, 2024

Reporting Summary

Nature Portfolio wishes to improve the reproducibility of the work that we publish. This form provides structure for consistency and transparency in reporting. For further information on Nature Portfolio policies, see our Editorial Policies and the Editorial Policy Checklist.

Statistics

For all statistical analyses, confirm that the following items are present in the figure legend, table legend, main text, or Methods section.

n/a	Confirmed	
☐	☒	The exact sample size (n) for each experimental group/condition, given as a discrete number and unit of measurement
☒	☐	A statement on whether measurements were taken from distinct samples or whether the same sample was measured repeatedly
☒	☐	The statistical test(s) used AND whether they are one- or two-sided<br>*Only common tests should be described solely by name; describe more complex techniques in the Methods section.*
☒	☐	A description of all covariates tested
☐	☒	A description of any assumptions or corrections, such as tests of normality and adjustment for multiple comparisons
☐	☒	A full description of the statistical parameters including central tendency (e.g. means) or other basic estimates (e.g. regression coefficient) AND variation (e.g. standard deviation) or associated estimates of uncertainty (e.g. confidence intervals)
☒	☐	For null hypothesis testing, the test statistic (e.g. F, t, r) with confidence intervals, effect sizes, degrees of freedom and P value noted<br>*Give P values as exact values whenever suitable.*
☒	☐	For Bayesian analysis, information on the choice of priors and Markov chain Monte Carlo settings
☒	☐	For hierarchical and complex designs, identification of the appropriate level for tests and full reporting of outcomes
☐	☒	Estimates of effect sizes (e.g. Cohen's d, Pearson's r), indicating how they were calculated

Our web collection on statistics for biologists contains articles on many of the points above.

Software and code

Policy information about availability of computer code

Data collection	Raw training data are sourced from public databases and procedures can be found in the manuscript.
Data analysis	Code to reproduce the experiments can be found at https://github.com/jinsenli/deepDNAshape and https://doi.org/10.5281/zenodo.10403299. Other software used in the production are PyMOL v2.5.4, Curves v5.3, X3DNA v2.2, DNAshapeR v1.30.0 and TensorFlow 2.11.1

For manuscripts utilizing custom algorithms or software that are central to the research but not yet described in published literature, software must be made available to editors and reviewers. We strongly encourage code deposition in a community repository (e.g. GitHub). See the Nature Portfolio guidelines for submitting code & software for further information.

Data

Policy information about availability of data

All manuscripts must include a data availability statement. This statement should provide the following information, where applicable:

- Accession codes, unique identifiers, or web links for publicly available datasets
- A description of any restrictions on data availability
- For clinical datasets or third party data, please ensure that the statement adheres to our policy

PDB id used in the main manuscript: 1AN2, 2R5Z and 4CYC. Raw training data and other datasets were sourced from previous publications or public databases (Li. et al., 2017, NAR.; PDB and ParmBSC1 forcefield Nucleotide MD Simulations Database https://mmb.irbbarcelona.org/ParmBSC1/) and procedures can be found in the manuscript, and are deposited in https://doi.org/10.5281/zenodo.10403307

Human research participants

Policy information about studies involving human research participants and Sex and Gender in Research.

Reporting on sex and gender	N/A
Population characteristics	N/A
Recruitment	N/A
Ethics oversight	N/A

Note that full information on the approval of the study protocol must also be provided in the manuscript.

Field-specific reporting

Please select the one below that is the best fit for your research. If you are not sure, read the appropriate sections before making your selection.

☒ Life sciences ☐ Behavioural & social sciences ☐ Ecological, evolutionary & environmental sciences

For a reference copy of the document with all sections, see nature.com/documents/nr-reporting-summary-flat.pdf

Life sciences study design

All studies must disclose on these points even when the disclosure is negative.

Sample size	We attempted to use as many training data as possible from 3 different data sources (MC, MD and Expt). For TF-DNA binding data, we always use the full dataset preprocessed by previous studies. In generating Figure 2e and 2f, sample size is 16,384 which represents the full dataset (all possible 7-mer).
Data exclusions	We excluded "irregular" B-DNA helical regions of DNA in experimentally solved structures, according to methods described in supplementary text. We excluded regions where DNA shape values cannot be derived in generating the training data.
Replication	Analysis and results were repeated multiple times. For example, Deep DNAshape models are trained multiple times during model design/ refinement stage and the performance is all better than previous studies.
Randomization	In hyper parameter searches for Deep DNAshape models, training data were split into training and test on 8/2 ratio. In multiple linear regression (MLR) models, data were split into 10-fold cross validation based on method in Li., et al., Nucleic Acids Research, 2017.
Blinding	The study is purely computational. To reduce bias, dropout layers and hyper parameter searches are used in training the Deep DNAshape model. Cross validation and L2-regularization is used in the MLR models.

Reporting for specific materials, systems and methods

We require information from authors about some types of materials, experimental systems and methods used in many studies. Here, indicate whether each material, system or method listed is relevant to your study. If you are not sure if a list item applies to your research, read the appropriate section before selecting a response.

Materials & experimental systems

n/a	Involved in the study
☒	☐ Antibodies
☒	☐ Eukaryotic cell lines
☒	☐ Palaeontology and archaeology
☒	☐ Animals and other organisms
☒	☐ Clinical data
☒	☐ Dual use research of concern

Methods

n/a	Involved in the study
☒	☐ ChIP-seq
☒	☐ Flow cytometry
☒	☐ MRI-based neuroimaging
